# Commonly available but highly effective protection against SARS-CoV-2 during gastrointestinal endoscopies

**DOI:** 10.1371/journal.pone.0254979

**Published:** 2021-07-23

**Authors:** Radan Keil, Štěpán Hlava, Petr Stanovský, Vladimír Ždímal, Jan Šťovíček, Milan Trojánek, Jiří Drábek, Barbora Frýbová, Vojtěch Petráček, Martin Wasserbauer

**Affiliations:** 1 Department of Internal Medicine, 2nd Faculty of Medicine Charles University in Prague and Motol University Hospital, Prague, Czech Republic; 2 Institute of Chemical Process Fundamentals of the Czech Academy of Sciences, Prague, Czech Republic; 3 1st Department of Infectious Diseases, 2nd Medical Faculty Charles University in Prague and Hospital Na Bulovce, Prague, Czech Republic; 4 Department of Pediatric Surgery, 2nd Faculty of Medicine Charles University in Prague and University Hospital Motol, Prague, Czech Republic; 5 Faculty of Nuclear Sciences and Physical Engineering, The Czech Technical University, Prague, Czech Republic; University of Malaya Faculty of Medicine, MALAYSIA

## Abstract

**Background and aims:**

SARS-CoV-2 is a worldwide serious health problem. The aim of this study was to demonstrate the number of potentially infectious particles present during endoscopic procedures and find effective tools to eliminate the risks of SARS-CoV-2 infection while performing them.

**Methods:**

An experimental model which focused on aerosol problematics was made in a specialized laboratory. This model simulated conditions present during endoscopic procedures and monitored the formation of potentially infectious fluid particles from the patient’s body, which pass through the endoscope and are then released into the environment. For this reason, we designed and tested a prototype of a protective cover for the endoscope’s control body to prevent the release and spread of these fluid particles from its working channel. We performed measurements with and without the protective cover of the endoscope’s control body.

**Results:**

It was found that liquid coming through the working channel of the endoscope with forceps or other instruments inside generates droplets with a diameter in the range of 0.1–1.1 mm and an initial velocity of up to 0.9 m/s. The average number of particles per measurement per whole measured area without a protective cover on the endoscope control body was 51.1; with this protective cover on, the measurement was 0.0, p<0.0001.

**Conclusions:**

Our measurements proved that fluid particles are released from the working channel of an endoscope when forceps are inserted. A special protective cover for the endoscope control body, made out of breathable material (surgical cap) and designed by our team, was found to eliminate this release of potentially infectious fluid particles.

## Introduction

At the end of 2019, a number of serious cases a previously unknown disease mainly affecting the respiratory tract (later known as Covid-19) were first reported in China. Soon after, the number of cases increased dramatically and infection expanded worldwide. The etiological agent of this disease has been confirmed as a novel coronavirus and named SARS-CoV-2. As of 1^st^ June 2021, more than 170 000 000 cases of the disease had been confirmed with over 3 500 000 deaths. As of this writing, these numbers continue to grow significantly day by day.

The SARS-CoV-2 pandemic is a serious and international health problem. This infection is characterized by rapid transmission of the virus between people. The basic reproduction number (R0) of SARS-CoV-2 is estimated at 1.4–2.5 [1], which is approximately twice as high as seasonal flu [2]. The main routes of transmission are via the respiratory route through inhalation of airborne droplets, conjunctival contact or via contact with infected secretions [3–5]. The virus has also been detected in samples of patient stool [6–8]. The most dangerous factor of this infection is transmission from people who are asymptomatic or have mild symptoms [9, 10].

SARS-CoV-2 is a significant health risk in terms of morbidity and also mortality to healthcare professionals (HCPs) who are at significantly higher risk of SARS-CoV-2 infection than the general population [11]. This risk is higher in HCPs with inadequate access to personal protective equipment [11, 12]. Some types of exposures (intubations, direct contact with secretions of the bodies) are associated with increased infection risk [12], which means that rapid and effective ways of protection are necessary to protect HCPs. Endoscopy of the upper gastrointestinal tract is a droplet/aerosol-generating procedure [13]. For HCPs working in the endoscopy department (as frontline HCPs), this procedure may lead to a higher risk of Covid-19 exposure (up to 10-fold increased risk of COVID-19 infection when compared to the general population) [11, 14] and is classified as a high-risk procedure in the matter of SARS-CoV-2 transmission. Infections of SARS-CoV-2 during gastrointestinal endoscopies have been reported [15–17].

Our hypothesis was that in routine clinical practice, fluid particles which are released during an endoscopic procedure, especially from the working channel of an endoscope, may be a potential source of spread in the context of the current SARS-CoV-2 pandemic. The primary goal of this study was to demonstrate the number of potentially infectious particles present during endoscopic procedures. The secondary goal was to find effective tools to eliminate the risks of SARS-CoV-2 infection to medical staff during endoscopic procedures by implementing some simple preventive processes against its spread. In cooperation with the Czech Academy of Sciences, we initiated the creation of an experimental model that simulated the pressure and other conditions present during an endoscopic procedure and monitored the formation of potentially infectious particles.

## Materials and methods

**Fig 1:**
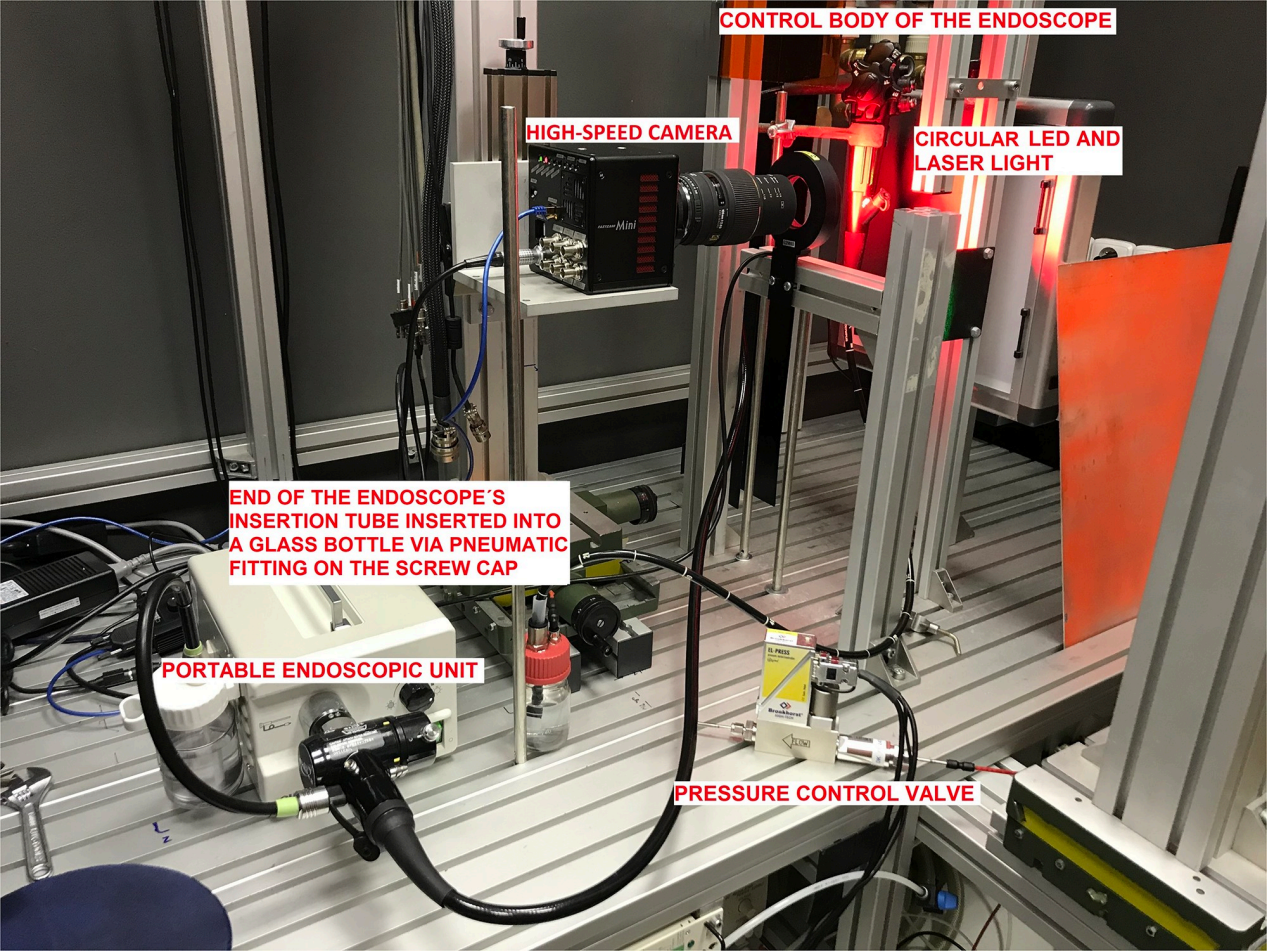
Experimental model simulating endoscopic examination. The picture shows our experimental model simulating endoscopic examination together with descriptions of its main components.

An experimental model simulating endoscopic examination (including pressure and other conditions) was designed (Fig 1). Videos were recorded with a high-speed camera (PhotronFastCam Mini AX200) at 250fps with an exposure of 1/1000 on a macro lens Sigma 105mm. Visualization was done at the instrument exit on the endoscope handle covering an area of 28 x 28 mm with front illumination via a circular LED light. Furthermore, laser light scattering was employed to identify small particles using a laser sheet with a thickness of 2mm (Quantronix Darwin, 527nm). The value of intragastric pressure was controlled via a forward pressure control valve (Bronkhorst) in the range of 2,5-50kPa installed in the laboratory on a 100ml glass bottle with a GL45 screw cap. The control body of the endoscope was firmly fixed in the rack and active suction by the endoscope was not used. The end of the endoscope was inserted into the vessel via a pneumatic fitting on the screw cap and was alternatively positioned above or into the liquid in the vessel (0.9% NaCl physiological saline solution). Droplet sizes and their velocities were analyzed via Photron PVF software.

During our measurements, a standard diagnostic front-view videogastroscope was used (Olympus CF-Q165I).

**Fig 2:**
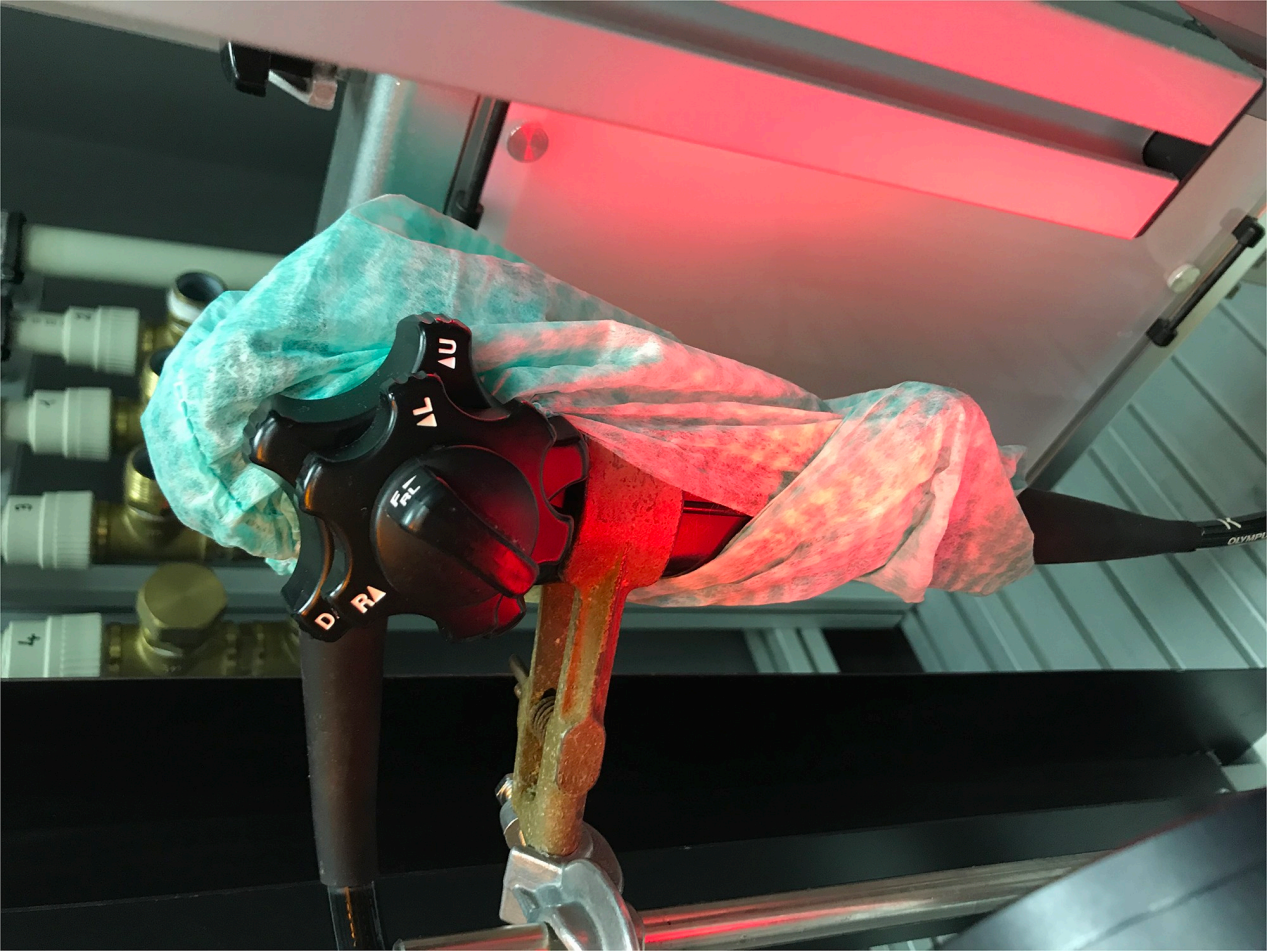
Protective cover. The picture shows a protective cover on an endoscope control body, which was made by wrapping the control body of the endoscope with a surgical cap.

Our measurements were focused on the excretion of water particles from the working channel of the endoscope. We performed the measurements using an experimental model in several situations and conditions. We performed the measurements in two main parts: with and without the protective cover of the endoscope’s control body. The protective cover was made from a commonly used and widely available surgical cap (specifically in our experiment, a Mölnlycke BARRIER surgical cap Kosack standard, REF 621000, was used) which was easily wrapped around the control body of the endoscope (Fig 2). The surgical cap is made from viscose nonwoven material. Other examined conditions were especially: whether the end of the endoscope was immersed in a fluid or not; the presence or non-presence of forceps in a working channel (forceps were not changed and only slight movements in and out were made during our measurements); the overpressure rate; and whether or not laser light is used for detection of aerosol or not.

One hundred images showing the release of fluid particles from the working channel of the endoscope were extracted from the recorded video—fifty with and fifty without the protective cover. Two photos were taken each second for 25 consecutive seconds. The number of released particles was counted in these images. The measurement area (field of view) was calculated at 7.3 cm2.

Statistical analysis:

Standard statistics were used to describe primary data. The statistical significance for the normality of the distribution of values was analyzed using a Shapiro-Wilk test; different datasets were compared using an unpaired T-test.

## Results

Several types of measurements were made (Table 1).

**Table 1:** Results of measurements.

Number of measurement	Presence of protective cover on the endoscope control body	Position of the end of the endoscope in glass bottle	Presence of forceps in a working channel	Overpressure rate (kPa)	Results (presence of fluid particles released from the working channel of the endoscope	Laser light (detection of aerosol)	Results (presence of aerosol released from the working channel of the endoscope
**1**	**NO**	**above the liquid level**	**YES**	**33**	**POSITIVE**	**NO**	
**2**	**NO**	**immersed in liquid**	**NO**	**33**	**NEGATIVE**	**NO**	
**3**	**NO**	**above the liquid level**	**YES**	**20**	**POSITIVE**	**NO**	
**4**	**NO**	**above the liquid level**	**YES**	**17–20**		**YES**	**NEGATIVE**
**5**	**NO**	**immersed in liquid**	**YES**	**50**		**YES**	**NEGATIVE**
**6**	**NO**	**immersed in liquid**	**NO**	**38–50**		**YES**	**NEGATIVE**
**7**	**NO**	**above the liquid level**	**YES**	**22**		**YES**	**NEGATIVE**
**8**	**NO**	**above the liquid level**	**YES**	**15–22**	**POSITIVE**	**NO**	
**9**	**NO**	**immersed in liquid**	**YES**	**30**	**NEGATIVE**	**NO**	
**10**	**YES**	**above the liquid level**	**NO**	**25–30**	**NEGATIVE**	**NO**	
**11**	**YES**	**above the liquid level**	**YES**	**9–10**	**NEGATIVE**	**NO**	
**12**	**YES**	**immersed in liquid**	**YES**	**25**	**NEGATIVE**	**NO**	

### 1. Measurements without a protective cover on the endoscope control body

Initially, it was proved that no fluid particles were released during measurement without the presence of forceps in the working channel.

**Fig 3:**
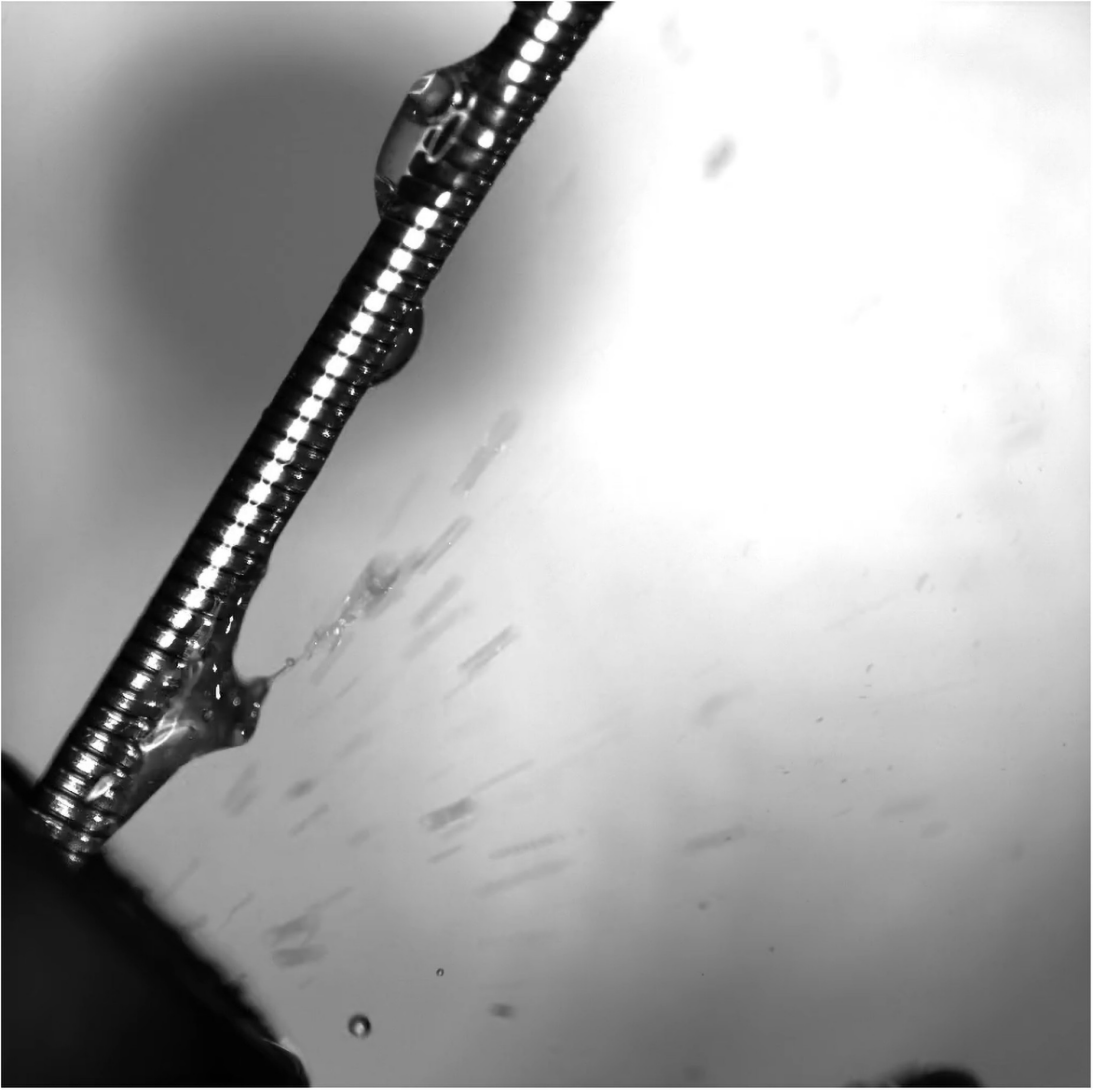
A release of fluid particles from the working channel of the endoscope. The picture shows generation and release of water droplets from the working channel of the endoscope when the forceps are inserted.

It was found that the liquid passing through the working channel of an endoscope with forceps or other instruments inside generate droplets with a diameter in the range of 0.1–1.1 mm and an initial velocity up to 0.9 m/s (Fig 3). The source of these droplets is an orifice where the instrument is inserted. A pocket of liquid and air carried through the instrument channel of the endoscope to form a plug which split into droplets of larger size. Especially during movement of an endoscopic instrument, bubbles are formed which burst into clouds of tiny droplets with high velocity, hence creating a large contamination area.

On the other hand, if the head of the endoscope is submerged in the liquid, the applied gas pressure delivers the liquid through the working channel only, causing the liquid to flow only along the handle or the instrument.

Illumination of the instrument exit on the endoscope handle via laser sheet with high light intensity in the dark did not show any presence of particles smaller than 0.03 mm, which can be considered as aerosol, and would require use of tighter covers with suitable filtration properties.

### 2. Measurements with a protective cover on the endoscope control body

**Fig 4:**
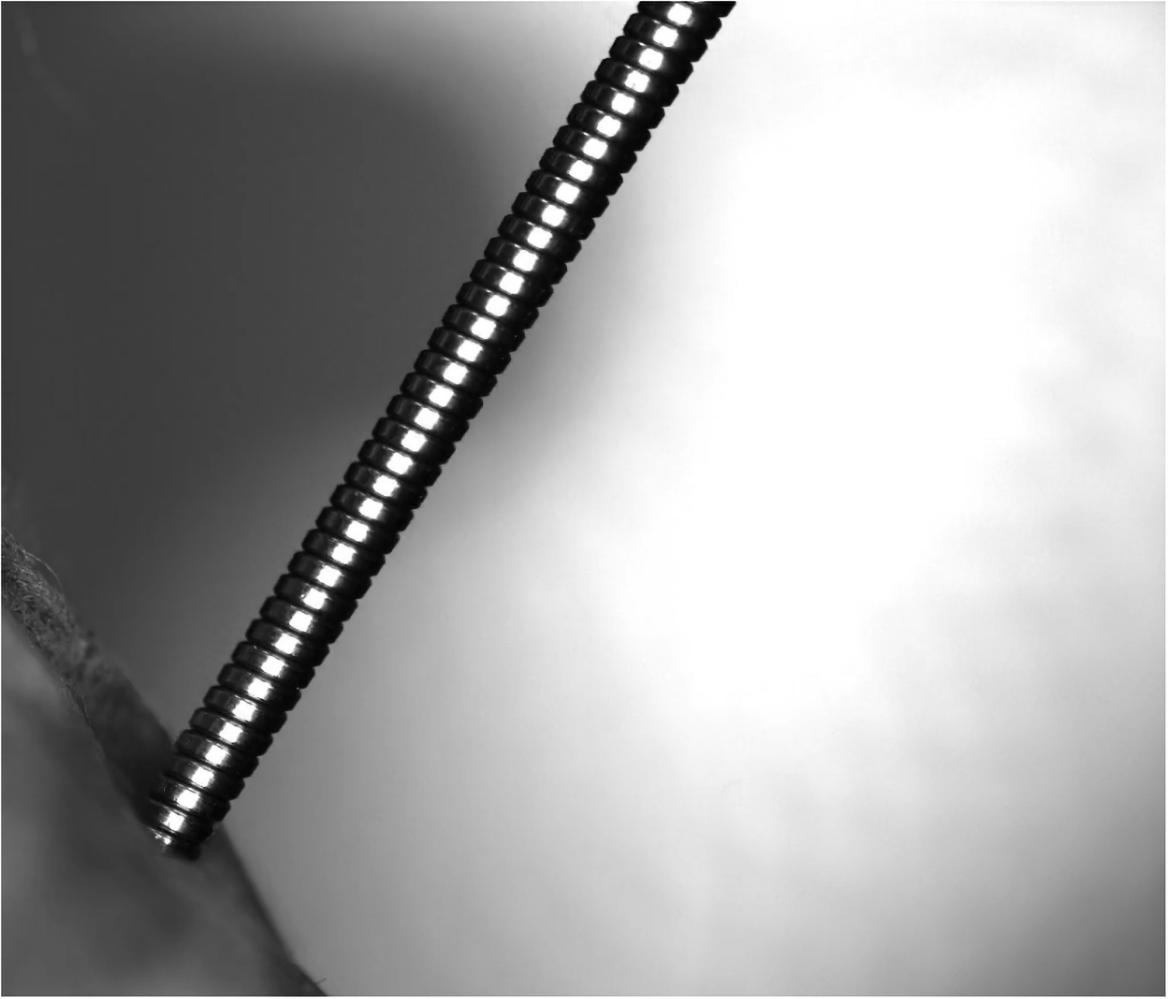
No release of fluid particles from the working channel of the endoscope. The picture shows that no water droplets are generated or released from the working channel thanks to the protective cover on the endoscope control body made out of a surgical cap.

During our measurements, it was proved that there is no release of fluid particles from the working channel of the endoscope (Fig 4). The release of fluid particles from the working channel was not affected by the position of the end of the endoscope in the glass bottle or by the presence of forceps in the working channel.

### 3. Counting of fluid particles in images

**Fig 5:**
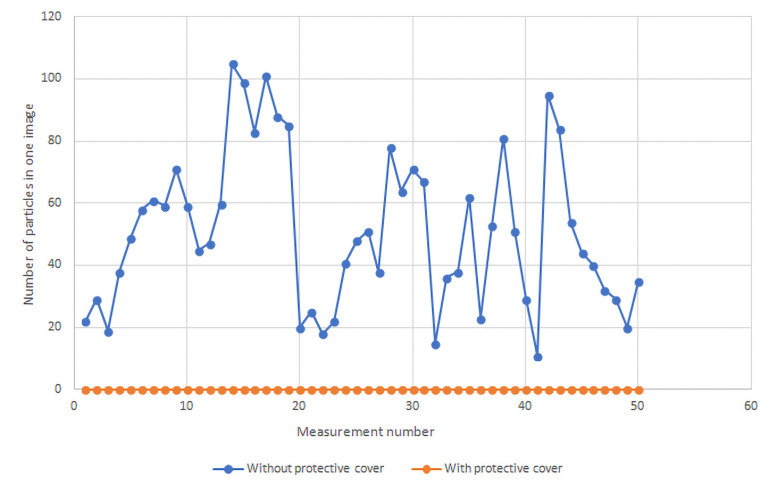
Number of released fluid particles. The picture shows a graph of released fluid particles from the working channel of the endoscope during our measurements (with and without the protective cover on the endoscope control body). No release of fluid particles was evident when the protective cover on the endoscope control body was used.

The average number of particles per measurement per whole measured area in fifty images for 25 consecutive seconds without the protective cover on the endoscope control body was 51.1, while with the protective cover the average number of particles was 0.0, p<0.0001 (Fig 5) (S1 Table).

The number of particles present per cm2 without the protective cover on the endoscope control body was 7.0 while with the protective cover the amount was 0.0, p<0.0001. The size of the observed released particles was 0.03–0.7 mm.

## Discussion

The main route of spread of SARS-CoV-2 is particularly via small droplets (usually between 5 μm to 10 μm) [18]. These particles of fluid are ejected primarily by speaking, coughing or sneezing [19]. High titers of SARS-CoV-2 are described in the saliva of patients with SARS-CoV-2 especially at the time of symptom presentation as well as in asymptomatic patients [20, 21]. Upper gastrointestinal endoscopic procedures are especially related to both aerosol generation and the spread of SARS-CoV-2 infection [17]. Subsequently, our study proved a release of fluid particles from the working channel of an endoscope, which moves from the tip throughout the entire length of the device when forceps or other endoscopic instruments are inserted.

**Fig 6:**
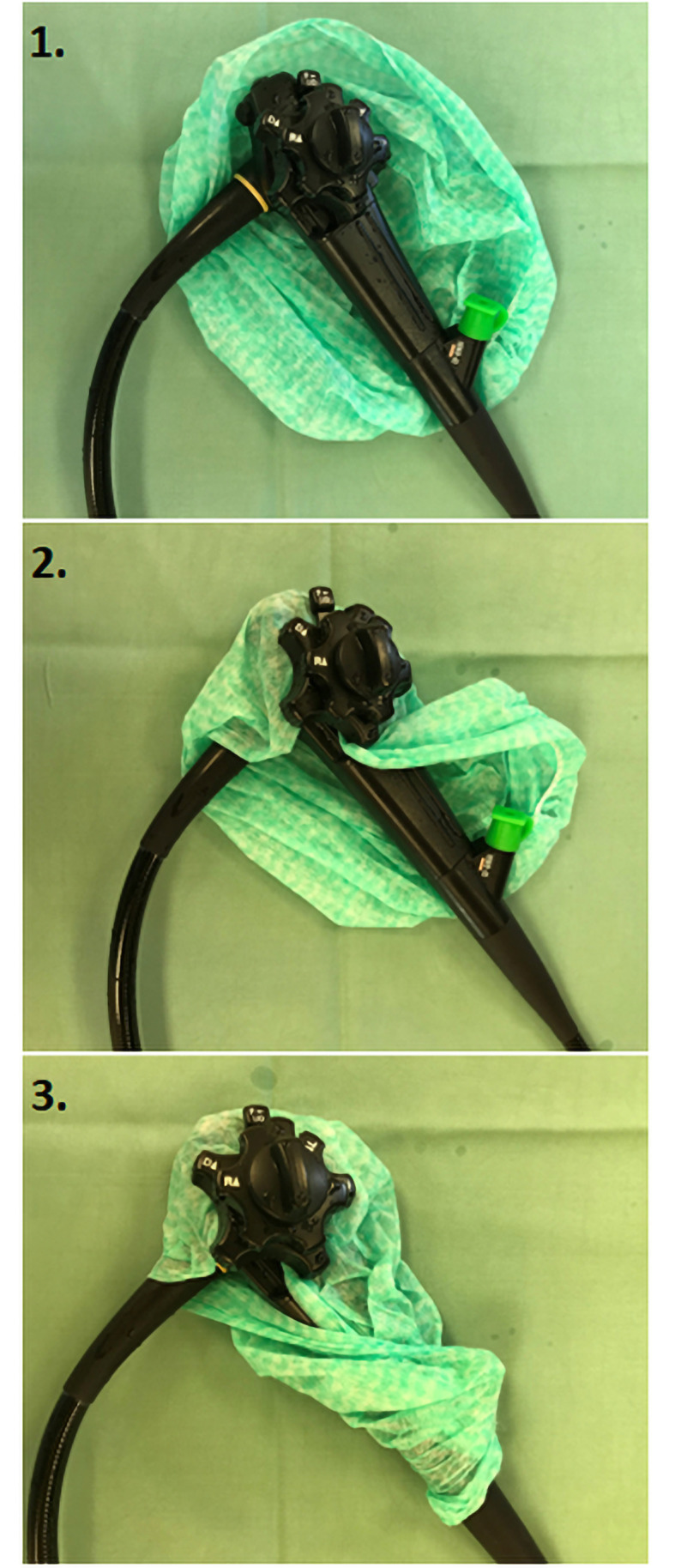
Instructions on how to create a protective cover. The picture shows instructions on how to create a protective cover for the endoscope control body in three steps.

Across all countries elective gastrointestinal endoscopic procedures were cancelled during the pandemic and only urgent endoscopies were performed [22]. Growing evidence about secondary consequences brought on by delays of diagnosis and treatment of gastrointestinal cancer are present [23]. There has been an increase in "pressure" regarding the necessity and administration of endoscopic procedures worldwide. Strategies aiming towards best practice of gastrointestinal endoscopies during the COVID-19 pandemic were made focusing mainly on patient triage and social distancing, personal hygiene and disinfection, prioritization of gastrointestinal endoscopic procedures, testing of SARS-CoV-2 and use of personal protective equipment [24, 25]. Gastroenterological/Endoscopy societies and teams are currently trying to assure the highest level of endoscopy care while also providing protection against SARS-CoV-2 at the same time. National as well as international guidelines (for example, ESGE guidelines) have been developed and research teams worldwide are trying to develop different types of personal protective equipment and protective techniques for administration of patient care. Methods of personal protection which have been developed and used include face shields and filtering face respirators, which are required to be worn by all personnel in the endoscopic unit regardless of patient status [16]. Other properties of personal protection include double pairs of gloves, eye protection with face shield, hairnets, shoe-covers and long-sleeve gowns with water resistance. The importance of wearing face masks among HCPs has been observed in three Chinese hospitals–among HCPs who were wearing face masks, no SARS-CoV-2 was manifested despite close contact with SARS-CoV-2 patients [14]. Face masks can be made of different materials and designs which influence their filtering capability. At least N95/FFP2 respirators are recommended for HCPs conducting aerosol-generating procedures during clinical care of SARS-CoV-2 patients or when performing high-risk procedures such as endoscopies [19]. Toner and Waldhorn point out that shortages of N95/FFP2 respirators should be anticipated, and if no other masks are available, surgical masks, which provide droplet protection, should be used instead [26]. We think that it is also necessary to find other additional ways to minimalize the release of potentially infectious particles, especially during high-risk medical procedures such as our study shows. This article provides you with instructions on how to create a protective cover for the endoscope control body in three steps (Fig 6).

With the idea of minimizing the spread of potential infectious fluid particles during endoscopic procedures, some technical improvements have been published. The conditions of these technical improvements must certainly be that it does not limit the necessary access to the patient and have no impact to the quality of the endoscopic procedure. Ljubicic and his colleagues have recently published a report on using a Plexiglas barrier box to improve ERCP safety during the SARS-CoV-2 pandemic [27] while de Grazia and his colleagues have published a report on the use of 3D printer-created face masks to protect endoscopy unit personnel in contact with COVID-19 high-risk patients [28].

We designed and created an experimental model simulating the conditions present during an endoscopy of the upper gastrointestinal tract under laboratory conditions. The model was created to simulate conditions arising during gastroscopy as realistically as possible. Tests were performed both with the tip of the endoscope above the fluid surface as well as immersed in the fluid (physiological saline solution was used), because both of these variants can be encountered during endoscopies. We also fixed the end of the endoscope to a glass bottle via a pneumatic fitting on the screw cap and created an overpressure in the glass bottle. The overpressure value was approximately correlated with the Iqbal and Haider study, which showed a maximum gastric overpressure during vomiting and retching of almost 300 mmHg [29]. On the other hand, we did not demonstrate the formation of an aerosol released from the working channel during an endoscopic procedure, as we had originally assumed. Aerosol formation and release is very likely to occur during endoscopy from the patient’s mouth. This hypothesis correlates with the conclusions of studies showing that aerosols and small droplets are already ejected during speaking, coughing or sneezing [19]. A very remarkable fact is the demonstration that only during the use of forceps or other endoscopic instruments is the release of fluid particles into the environment present at a fairly high speed and in large quantities. It is also necessary to keep in mind that endoscopic procedures require short distances between an endoscopist and his/her patients. A pocket of liquid and air carried through the instrument channel of the endoscope forms a plug which splits into droplets of larger size. When moving an endoscopic instrument, bubbles are formed which burst into clouds of tiny droplets with high velocity, hence creating a larger contamination area. This is in accordance with studies done on the role of bubbles and droplets in disease transmission in the environment [30]. The presence and use of instruments therefore significantly increase the risk in terms of possible transmission of SARS-CoV-2.

A study based on quantifying the rate of unrecognized exposure of potentially infectious biologic samples during endoscopy to the endoscopist’s face was published [31], which resulted in the need for universal facial protection during gastrointestinal endoscopy. Several variable factors may play a crucial role in the exposure of HCPs to potentially infectious material. These include place of examination of the gastrointestinal tract with different intraluminal pressures (gastroscopy, colonoscopy,…) as well as technical conditions (excessive using of suction, multiple exchanges of different types of devices [32]. These factors may increase the splash rate, which could affect the rate of exposure to HCPs. These data illustrate the importance of using barrier protection (especially keeping a safe distance from the patient and using personal protective equipment) in HCPs [33]. Our project may help to supplement this spectrum of protective aids in gastrointestinal endoscopy.

Fifty images showing the release of fluid particles from the working channel of the endoscope, with no protective cover present, were made. A total of 2 555 fluid particles released from the endoscope working channel were documented. The measured area was only about 7 cm2. If a larger area were measured, several times more fluid particles would certainly be measured. It should also be added that the particles were measured in only one plane. If we were able to capture all the particles in the whole 3D space, several times more fluid particles would also be measured. SARS-CoV-2 is an enveloped virus of 100 nm in diameter [34]. The size of the observed released particles were 0.03–0.7 mm. Thus, 300–7 000 virions could be contained in one plane of one fluid particle in the particles we observed. For many viruses, including SARS-CoV-2, even a small dose of virions can lead to serious infection.

Furthermore, our work has shown that the protective cover of the endoscope must be prepared exclusively from breathable materials. At first, experts from the Czech Academy of Sciences rejected our proposed solution for the use of an airtight material (eg, a microtene bag) due to the fact that the use of this material would only redirect the air flow with potentially infectious fluid particles. When a breathable material is used, potentially infectious particles are trapped directly into the material. The efficiency of trapping these particles of different sizes is determined by the type of breathable material. The effectiveness of breathable materials (surgical masks) in preventing the spread of viral infections has been demonstrated in numerous studies [35, 36]. Also, technically this variant is better, as there is no obturation of the suction channel with impermeable material and, consequently, no permanent suction of the endoscope.

Limitations of this study are:

1. Differences in filtration properties of individual surgical caps according to domestic manufacturers. However, we do not expect great clinical difference compared to our result.
2. Our measurements were focused only on the release of potentially infectious fluid particles from the area of the working channel on the endoscope control body. Other parts of the endoscope were not examined in our study. However, thanks to our protective cover, the entire endoscope control body is covered. We hypothesize that if potentially infectious fluid particles are released from the area of the endoscope control body (especially suction valve and air / water valve), the protective cover will be also be able to eliminate them.
3. Some conditions of our measurement: no measurements with active intermittent suction of the endoscope were performed, no different types of instruments were used during our measurements (only one type of forceps) and during the measurements the forceps were not actively and completely pulled out and inserted (only small movements with the instruments were performed).
4. Excretion of potentially infectious particles is also possible through the patient’s mouth. We recommend covering the head of high-risk patients with breathable materials to eliminate especially large-volume droplets, which represent the largest viral load and thus a greater risk for infection of SARS-CoV-2.
5. Our measurements only simulated conditions during upper gastrointestinal tract endoscopy.
6. Our study was performed only as an experimental model.

## Conclusion

Our measurements proved that fluid particles are released from the working channel of an endoscope when forceps are inserted with a diameter in the range of 0.1–1.1 mm and an initial velocity up to 0.9 m/s. A special protective cover of the endoscope control body which is made out of breathable material (surgical cap) and designed by our team is able to eliminate this release of potentially infectious fluid particles. Thus, an effective and inexpensive way of protection during endoscopic procedures of the gastrointestinal tract with all of the relevant health and economic benefits for doctors, HCPs, and patients was developed.

## Supporting information

S1 Table Exact number of releasing fluid particles during our measurements per 1 field. The table shows quantities of released fluid particles from the working channel of the endoscope during our measurements (with and without the protective cover on endoscope control body). The measurement lasted 25 seconds, 2 images were evaluated in each second. All fluid particles of different sizes were then counted on each image.
(XLSX)

S1 Data (XLSX)
